# Live imaging of the genetically intractable obligate intracellular bacteria *Orientia tsutsugamushi* using a panel of fluorescent dyes

**DOI:** 10.1016/j.mimet.2016.08.022

**Published:** 2016-11

**Authors:** Sharanjeet Atwal, Suparat Giengkam, Michael VanNieuwenhze, Jeanne Salje

**Affiliations:** a Mahidol-Oxford Tropical Medicine Research Unit, Faculty of Tropical Medicine, Mahidol University, Bangkok, Thailand; b Centre for Tropical Medicine and Global Health, Nuffield Department of Medicine, University of Oxford, Oxford, United Kingdom; c Indiana University, Bloomington, IN, USA

**Keywords:** Fluorescence microscopy, Intracellular bacteria, Fluorescent dyes, Rickettsia, Neglected bacterial pathogens

## Abstract

Our understanding of the molecular mechanisms of bacterial infection and pathogenesis are disproportionally derived from a small number of well-characterised species and strains. One reason for this is the enormous time and resources required to develop a new organism into experimental system that can be interrogated at the molecular level, in particular with regards to the development of genetic tools. Live cell imaging by fluorescence microscopy is a powerful technique to study biological processes such as bacterial motility, host cell invasion, and bacterial growth and division. In the absence of genetic tools that enable exogenous expression of fluorescent proteins, fluorescent chemical probes can be used to label and track living cells. A large number of fluorescent chemical probes are commercially available, but these have overwhelmingly been applied to the study of eukaryotic cell systems. Here, we present a methodical analysis of four different classes of probes, which can be used to delineate the cytoplasm, nucleic acids, cell membrane or peptidoglycan of living bacterial cells. We have tested these in the context of the important but neglected human pathogen *Orientia tsutsugamushi* but expect that the methodology would be broadly applicable to other bacterial species.

## Highlights

• A panel of fluorescent dyes are used to label living cells of the obligate intracellular bacterium *Orientia tsutsugamushi*.
• These dyes include CFSE, SYTO9, DiI/DiO/DiD and HADA.
• These dyes label the bacterial cytoplasm, nucleic acids, lipid membrane and cell wall respectively.

## 1 Introduction

Fluorescence microscopy is a key tool in the study of bacterial cell biology and host-pathogen interactions. A multitude of different fluorescent proteins have now been developed, and these enable both the tracking of bacterial populations over time, and the tagging of specific proteins of interest to study their subcellular localisation and dynamics (Landgraf et al., 2012, Shaner et al., 2008). These powerful approaches, however, are limited to those bacterial species for which genetic manipulation is possible. Relative to the wealth and diversity of known bacterial species, the number of genetically tractable model organisms is miniscule. Fluorescent proteins are also limited by their inability to label molecular species that are not directly genomically encoded, such as the peptidoglycan cell wall and lipid bilayers. Small fluorescent probes that are able to diffuse into living cells and label specific components are powerful alternative tools. A large collection of probes have been developed for use in mammalian cells, including those with the ability to label generic molecules such as free amines or lipid bilayers (Brown and London, 1998), and those that target specific cellular structures such as lysosomes (Pol et al., 2001, Peng et al., 2010), the endoplasmic reticulum, or the actin or microtubule cytoskeleton (Belin et al., 2014). Comparatively few have been developed specifically for bacterial targets with the notable exception of peptidoglycan (Shieh et al., 2014, Kuru et al., 2015). Many of the probes developed for use in mammalian cells have not been tested in bacteria (Boleti et al., 2000).

An ideal fluorescent probe would be incorporated into cells using a minimally invasive protocol, would be photostable and long-lasting, would exhibit minimal detachment from its molecular target and have little or no effect on bacterial viability. All criteria may not always be fulfilled, and certain compromises may be acceptable depending on the specific scientific question being addressed.

*Orientia tsutsugamushi* is an obligate intracellular bacterium, and the mite-borne causative agent of the severe human disease scrub typhus (Watt and Parola, 2003, Rajapakse et al., 2012, Seong et al., 2001). Whilst this disease has an untreated mortality rate of between 1 and 40%, and is predicted to affect at least 1 million people annually (Watt and Parola, 2003), comparatively little is known about its mechanisms of host cell invasion and pathogenesis (Paris et al., 2013). There are currently no genetic tools available, and like all obligate intracellular bacteria, such as the *Chlamydiales* and the *Rickettsiales*, these organisms are osmotically sensitive and experimentally difficult to manipulate. The availability of experimentally validated fluorescent probes to label *O. tsutsugamushi* would enable live cell imaging experiments to study its host cell infection cycle in detail. Here, we have tested the application of a panel of fluorescent probes in *O. tsutsugamushi* and discuss their suitability for future cell biology experiments.

## 2 Results and discussion

### 2.1 Carboxyfluorescein succinimidyl ester (CFSE) can be used to label the cytoplasm of *O. tsutsugamushi*

Carboxyfluorescein succinimidyl ester is a fluorescent dye that is used to label mainly proteins in the cytoplasm of living cells. The non-fluorescent compound carboxyfluorescein diacetate succinimidyl ester (CFDA-SE) is added to living cells, and diffuses freely across the cell membrane (Bronner-Fraser, 1985, Nose and Takeichi, 1986, The Molecular Probes Handbook, n.d). Intracellular esterases cleave the two acetate groups to form fluorescent CFSE, which is not membrane-permeable and is thus confined to the cytoplasm. This compound reacts to form a covalent bond with lysines and other primary amines via its succinimidyl group, resulting in covalently labelled fluorescent proteins in the cellular cytoplasm. CFSE is extremely photostable and is commonly used as a long-term cell tracer in immunological studies of migration and proliferation in lymphocytes (Parish, 1999, Parish and Warren, 2002), and occasionally in experiments with bacteria (Obradovic et al., 2016, Tuominen-Gustafsson et al., 2006). Various derivatives of CFSE have been developed with different fluorescent properties, including a Far Red variant, which is used in this study.

**Fig. 1:**
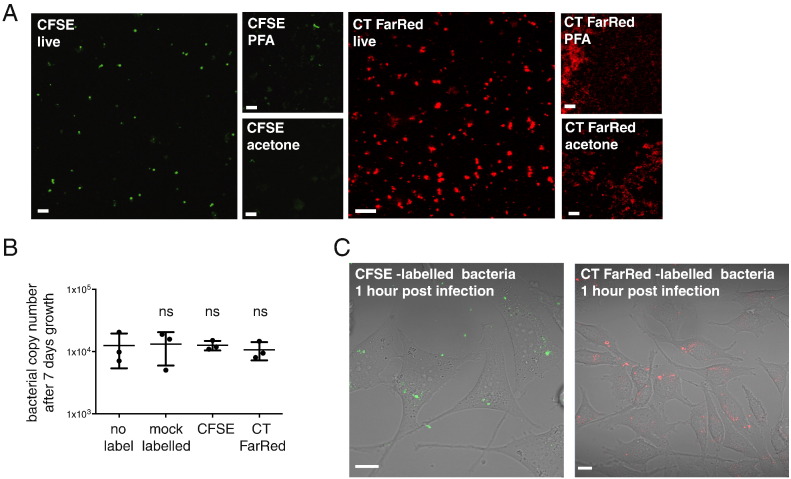
(A) Purified bacteria labelled with CFSE (left panels, green) or CellTrace FarRed (right, red) and imaged live or fixed with paraformaldehyde (PFA) or acetone prior to imaging. (B) Graph showing the bacterial copy number in one well of a 24-well culture plate after 7 days growth in the presence or absence of CFSE/CT FarRed. Inoculum used was 1.36 × 10^4^ ± 1.9 × 10^3^ bacteria per well (mean ± SD). Individual replicate values are plotted and the mean ± SD is shown. The results of the one-way ANOVA statistical test using the no label condition as a comparison are shown. (C) Images of live L929 cells infected with CFSE or CT FarRed-labelled bacteria. Scale bar = 10 μm. (For interpretation of the references to colour in this figure legend, the reader is referred to the web version of this article.)

We tested the ability of (green) CFSE and (red) CellTrace FarRed (CT FarRed) to label *O. tsutsugamushi* cells and found that the bacteria could be clearly labelled with the dyes at 5 μM (Fig. 1A). *O. tsutsugamushi* labelled with these dyes appeared as circles or coccobacilli of around 1–2 μm in diameter, which would be expected from cytoplasmic labelling of these cells. These dyes are reported to be amenable to fixation with aldehydes (Molecular Probes, USA) but in our hands the labelling could not be properly retained after fixation with either paraformaldehyde or acetone (Fig. 1A). We measured the effect of CFSE and CT Far Red on the growth of *O. tsutsugamushi* and found no detectable reduction in growth after 7 days compared with untreated and mock-treated bacteria (Fig. 1B and Supplementary Fig. 1). We tested whether we could follow bacterial attachment and entry into host cells using these dyes, and found that CFSE- and CT FarRed-labelled bacteria could be clearly observed when added to a monolayer of mammalian cells (Fig. 1C and Movie 1). Labelling with these dyes is performed on bacteria that have been isolated from host cells, and residual CFDA-SE is washed away after labelling. Therefore the fluorescent background in host cells from non-specific CFSE labelling is negligible. These results show that these dyes can be used to track the early events of bacteria attachment and entry.

### 2.2 CFSE can be used to follow bacterial division

**Fig. 2:**
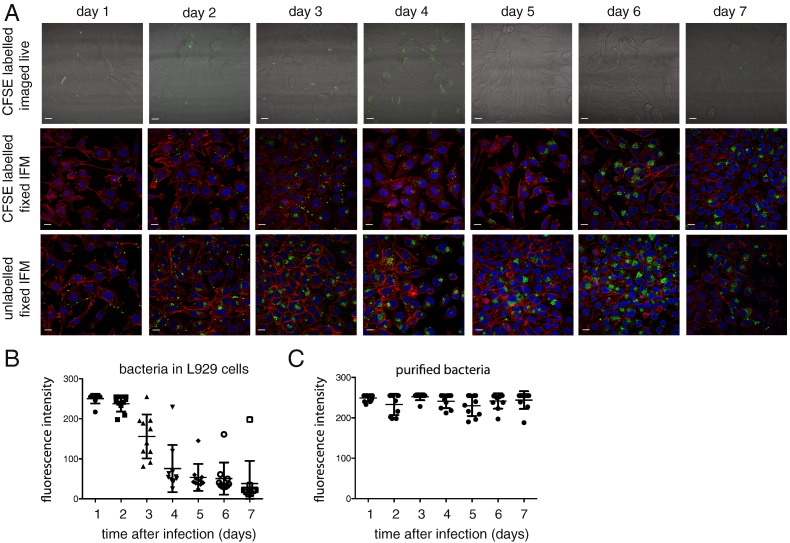
(A) Time course showing L929 cells infected with CFSE-labelled or unlabelled bacteria at various times after infection. The top row shows images of live L929 cells infected with CFSE-labelled bacteria. These images were not taken under equivalent imaging conditions (laser strength and acquisition time). The middle and lower rows show samples that have been fixed with paraformaldehyde and labelled with a fluorescently labelled antibody against *Orientia* TSA56 outer membrane protein (green, bacteria) or the actin-binding dye phalloidin (red, L929 cells). Scale bar = 10 μM. (B) and (C) Pixel intensity of CFSE-labelled bacteria stored in media (B) or added to L929 cells (C) at various times after labelling and infection. Images for this analysis were obtained under constant imaging conditions. (For interpretation of the references to colour in this figure legend, the reader is referred to the web version of this article.)

The covalent attachment of CFSE to cytoplasmic free amines means that the dye should be stably maintained within the cytoplasm and any reduction in fluorescence intensity should be largely due to cell division. The decrease in fluorescence intensity over time can therefore be used to quantify cell division. This principle has been used to track motility and proliferation of immune cells using CFSE (Parish, 1999, Parish and Warren, 2002), and also cell division and differentiation into non-dividing persistor cells in salmonella using genomically-encoded fluorescent proteins (Helaine et al., 2010). In order to determine whether CFSE could be used to quantify bacterial cell division in *O. tsutsugamushi* over time we quantified the fluorescence intensity of individual CFSE-labelled bacterial cells over 7 days. Separate glass slides with coverslip bases were used for each time point, in order to avoid reductions in fluorescence due to photobleaching from previous measurements or effects on bacterial growth due to extended periods of time on the microscope incubation chamber. In order to confirm that CFSE-labelling had no effect on bacterial morphology or the infection cycle, we performed this time course in parallel with samples that were fixed and immunolabelled using conventional methods. We fixed samples that had either been CFSE-labelled, or unlabelled. Fig. 2A shows a reduction in CFSE fluorescence over time (top row). The laser was increased in order to optimise image acquisition in these images, but during acquisition of images for quantification of fluorescence intensity the laser strength and gain parameters were kept constant. The middle row and lower row show a typical field of infected cells at each time point, where bacteria had (middle row) or had not (lower row) been labelled with CFSE. The estimated multiplicity of infection (MOI) was 100:1 bacteria:host but it should be noted that due to difficulties in rapid quantification of isolated *Orientia* this could not be measured precisely and there were unavoidable variations in MOI between conditions. These experiments were only used to perform qualitative analysis of bacterial growth. The infection cycle of *O. tsutsugamushi* is known to involve translocation to the nucleus after 24–48 h followed by bacterial replication. The patterns of bacterial localisation and replication in both time courses were comparable, and similar to large numbers of other time courses performed in our laboratory with this strain and cell line. Therefore we concluded that CFSE labelling did not significantly affect the typical infection cycle of *O. tsutsugamushi*.

At each time point images of live bacteria were acquired and 10 bacteria were randomly selected for quantification. The highest pixel intensity within each bacterium (which typically covered ~ 5–10 pixels) was measured and recorded. This experiment was performed 3 separate times. As a comparison, CFSE-labelled bacteria that were not added to host cells and therefore not able to divide were quantified in the same way. Fig. 2B shows that average the fluorescence intensity of *O. tsutsugamushi* decreased rapidly between 48 and 96 h. In comparison, bacteria that had been stored in the absence of host cells showed no decrease in fluorescence (Fig. 2C) indicating that the reduction in fluorescence was due to bacterial growth and division rather than reduction in fluorescence intensity of the probe. The timing of the decrease in CFSE fluorescence correlates closely with the known replication time of *O. tsutsugamushi* as measured by qPCR (Giengkam et al., 2015). One limitation of qPCR is that it measures genome copy number rather than cell number. Since it is known that some bacteria can carry more than one genome copy per cell this may not be an accurate representation of cell division, and the decrease in CFSE intensity offers an independent measure of bacterial replication.

A small number of bacteria with high CFSE levels could be detected up to 7 days after infection (Fig. 2B), indicating a sub-population of cells that are either dead or only slowly dividing. This may represent a persistent population as has been reported for other intracellular bacteria (Helaine et al., 2014). One limitation of our approach is that we cannot distinguish between reductions in fluorescence due to bacterial division and reductions due to secretion of effector proteins. Whilst we expect the total number of secreted proteins as a factor of the total proteome to be small enough not to affect the cytoplasmic CFSE level dramatically, this has not been experimentally verified.

### 2.3 SYTO9 can be used to label nucleic acids in live *O. tsutsugamushi*

**Fig. 3:**
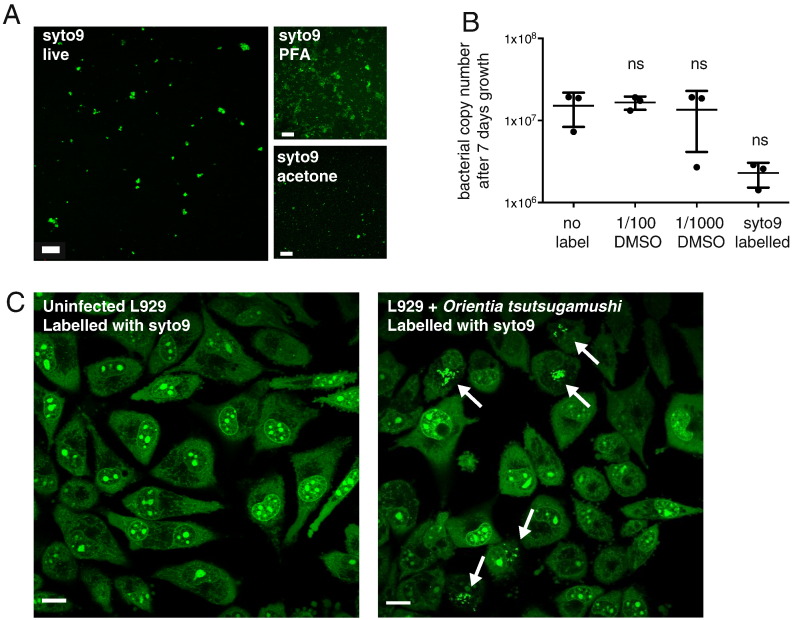
(A) Purified bacteria labelled with the green DNA-binding dye SYTO9 and imaged live or fixed with paraformaldehyde (PFA) or acetone prior to imaging. (B) Graph showing the bacterial copy number in one well of a 24-well culture plate after 7 days growth in the presence or absence of DMSO or SYTO9. Inoculum used was 4.09 × 10^5^ ± 1.8 × 10^4^ bacteria per well (mean ± SD). Individual replicate values are plotted and the mean ± SD is shown. The results of the one-way ANOVA statistical test using the no label condition as a comparison are shown. (C) Images of live L929 cells infected with SYTO9-labelled bacteria. White arrows indicate bacteria. Scale bar = 10 μm.

SYTO9 is a green-fluorescent dye that is freely permeable to cell membranes and undergoes a large increase in fluorescence upon binding to nucleic acids. SYTO9 is a component of the popular live/dead viability kit, in which it is combined with a second nucleic acid binding dye, propridium iodide, that cannot cross intact membranes and therefore only labels dead cells in which the membrane is disrupted (Maurer and Dougherty, 2001). We tested whether SYTO9 could be used to label intact, live *O. tsutsugamushi* cells. We found that addition of 5 nM SYTO9 resulted in bright and clearly labelled bacterial cells, which could be observed almost immediately after addition (Fig. 3A). The labelling procedure was extremely straightforward, with DMSO-solubilised probe being added directly to living cells. Since the dye is not fluorescent until bound to nucleic acids, further washing steps are not required. Similar to CFSE and CT FarRed, we found that SYTO9 fluorescence was not retained after fixation with paraformaldehyde or acetone (Fig. 3A). We tested the effect of SYTO9-labelling on bacterial growth and found that it resulted in a reproducible reduction in growth over 7 days, although this was not statistically significant (Fig. 3B). We also determined the effect of DMSO on bacterial growth, since this is a common solvent for many chemical compounds, and found no effect on bacterial growth when added at a dilution of 1/100 or 1/1000 (Fig. 3B). The concentration used in the SYTO9 labelling was 1/1000.

*O. tsutsugamushi* exhibits a long cellular infection cycle *in vitro*, lasting 7 days or more (Seong et al., 2001, Moree and Hanson, 1992). Whilst CFSE provided a powerful tool for monitoring the early stages of bacterial attachment and entry, the fact that bacteria could only be labelled at the start of infection made it difficult to study processes occurring after one or two rounds of bacterial division using this dye. We therefore wondered whether we could use SYTO9 to label bacteria inside cells that had already been infected for several days. This would enable studies of bacterial localisation and motility during periods of rapid replication and host cell exit. SYTO9 labels nucleic acids non-discriminately and therefore host cell nuclei, mitochondria and cytoplasmic RNA will also be labelled. In spite of this we found that fluorescently labelled *O. tsutsugamushi* could be clearly identified against this background due to their number, perinuclear localisation and rapid motility (Fig. 3C and Movie 2). Given the observed effect on bacterial replication over time, this dye is not suitable for long term imaging studies. However, it promises to be a powerful tool in the study of motility of intracellular pathogens. It also provides an alternative and rapid method for the quantification of bacteria in infected cultured cells.

### 2.4 The DiI, DiO and DiD vybrant dyes can be used to label the membrane of *O. tsutsugamushi*

Clear labelling of bacterial membranes enables analysis of bacterial cell shape as well as a study of the formation of outer membrane vesicles. The styryl dye FM 4–64 is commonly used to label the membranes of bacterial cells (Pogliano et al., 1999), but labelling efficiency is species-specific and we found it labelled *O. tsutsugamushi* poorly (data not shown). Furthermore, whilst this dye only exhibits fluorescence when inserted in a lipid bilayer, it has the property of reversibly diffusing in and out of membranes, resulting in a secondary labelling of nearby cells (Betz and Bewick, 1992). This inability to be retained within a specific subpopulation of cells limits it use in host-pathogen cell biology experiments. The vybrant dye collection comprises a range of highly lipophilic compounds that exhibit a range of fluorescent colours upon insertion into lipid membranes (Molecular Probes, USA). Unlike the FM collection, they exhibit lateral diffusion within their target membrane but no soluble diffusion to nearby cells in which membranes are not contiguous (The Molecular Probes Handbook, n.d.). This property makes it possible to label a distinct population of cells at the beginning of an experiment and follow this population over time.

**Fig. 4:**
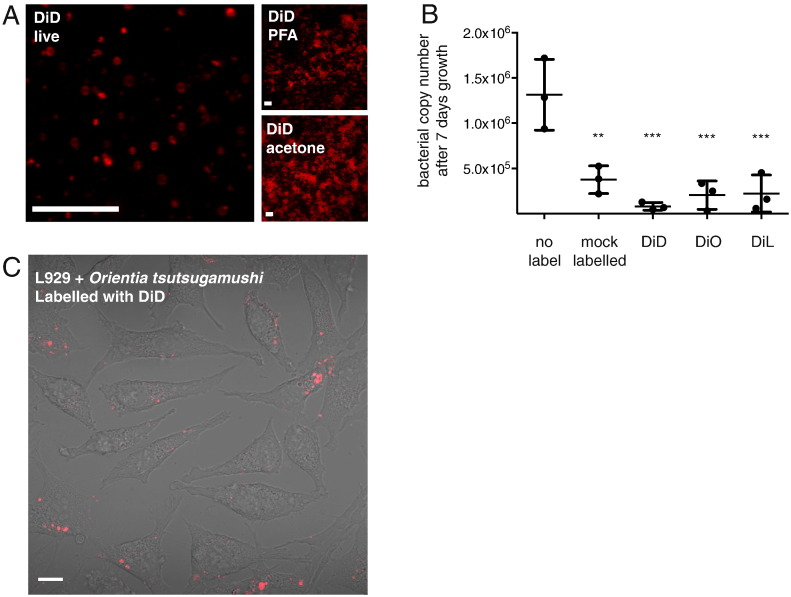
(A) Purified bacteria labelled with the red membrane-binding dye DiD and imaged live or fixed with paraformaldehyde (PFA) or acetone prior to imaging. (B) Graph showing the bacterial copy number in one well of a 24-well culture plate after 7 days growth in the presence or absence of DiD, DiI and DiL. Inoculum used was 1.5 × 10^5^ ± 7.7 × 10^3^ bacteria per well (mean ± SD). Individual replicate values are plotted and the mean ± SD is shown. The results of the one-way ANOVA statistical test using the no label condition as comparison are shown. **P ≤ 0.01; ***P ≤ 0.001. (C) Images of live L929 cells infected with DiD-labelled bacteria. Scale bar = 10 μm.

We found that *O. tsutsugamushi* could be clearly labelled with the vybrant dye DiD, DiI and DiO, revealing a range of bacterial structures (Fig. 4A shows bacteria labelled with DiD; DiI and DiO gave similar patterns of labelling). DiD labelling was not retained after fixation with paraformaldehyde or acetone. The procedure for DiD labelling involves resuspension of cells in a specific buffer (optimem) and we found that this affected bacterial growth over time (Fig. 4B). This effect could potentially be mediated by the development of alternative buffers. The additional reduction in bacterial growth caused by the vybrant dyes added at 100 μM was minimal (Fig. 4B). When membrane-labelled *O. tsutsugamushi* was added to unlabelled host cells there was no detectable background and the bacteria could be followed during attachment and entry into host cells (Fig. 4C). One potential application of this dye is in the study of generation and localisation of bacterial-derived outer membrane vesicles.

### 2.5 HADA can be used to label the peptidoglycan cell wall of *O. tsutsugamushi*

Peptidoglycan is a bacteria-specific molecule that is essential for most bacteria and that confers cell strength and rigidity (Turner et al., 2014, Vollmer et al., 2008) It is typically composed of a large structure that is positioned between the two membranes of Gram negative bacteria or outside the cell membrane of Gram positive bacteria, and that is comprised of a polymerised glycan backbone cross-linked through short peptide side chains. *O. tsutsugamushi* has always been reported to lack this macromolecule (Amano et al., 1987), but we recently showed that it is indeed present in these cells (manuscript submitted). A number of probes have been developed that specifically label bacterial peptidoglycan and these are based on fluorescent derivatives of the peptidoglycan-binding drug vancomycin (Turner et al., 2013), or the peptidoglycan-unique amino acid D-alanine (Kuru et al., 2015). Of these, the HCC-amino-D-alanine (HADA) probe can be used to image live cells and therefore we used this in the current study.

**Fig. 5:**
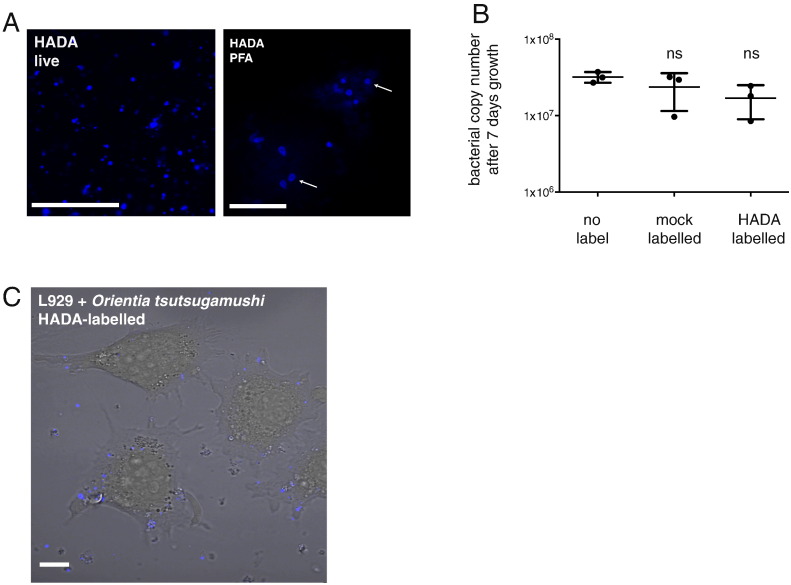
(A) Purified bacteria labelled with the blue peptidoglycan-binding dye HADA and imaged live or fixed with paraformaldehyde (PFA). Arrows point to bacteria where the HADA has labelled as a clear ring (B) Graph showing the bacterial copy number in one well of a 24-well culture plate after 7 days growth in the presence or absence of HADA. Inoculum used was 3.12 × 10^6^ ± 7.6 × 10^4^ bacteria per well (mean ± SD). The results of the one-way ANOVA statistical test using the no label condition as comparison are shown. (C) Images of live L929 cells infected with HADA-labelled bacteria. Scale bar = 10 μm.

Addition of HADA to *O. tsutsugamushi* yielded blue spots (live imaging, Fig. 5A, C and Movie 3) or clearly discernable rings corresponding to the bacterial cell wall (PFA-fixed, Fig. 5A). There was a high background from host cells as has been reported previously (Kuru et al., 2015) Unlike the other probes reported here, HADA-labelled *O. tsutsugamushi* could be fixed with paraformaldehyde, which will enable correlative studies combining live and fixed microscopy. We measured the growth of *O. tsutsugamushi* in the presence of HADA and found no significant effect on bacterial growth (Fig. 5B).

## 3 Conclusion

Here we describe the use of fluorescent dyes to label four distinct compartments of living bacterial cells: the cytoplasm, nucleic acids, cell membrane and the cell wall (summarised in Table 1). The ability to track living cells over time will enable the study of processes such as bacterial attachment and entry into host cells, bacterial cell division over time, and bacterial cell motility. The ability to label different components of the bacterial cell will enable the analysis of fundamental processes in bacterial cell biology such as growth and elongation, cytokinesis, and the generation of outer membrane vesicles. The methods described here will allow host-pathogen cell biology experiments to be performed in poorly characterised model systems, typical of neglected and emerging pathogens, fastidious organisms, or newly-isolated clinical or environmental strains.

## 4 Materials and methods

### 4.1 Cell culture and bacterial growth

All experiments were performed using the Karp-like UT76 strain of *Orientia tsutsugamushi* and the mouse fibroblast cell line L929. L929 cells were grown in 25 cm^2^ plastic flasks at 37 °C and 5% CO_2_, using DMEM or RPMI media supplemented with 10% FBS. For all experiments bacteria were isolated from L929 cells 7 days after infection and purified as described previously (Giengkam et al., 2015). Briefly, the supernatant was removed, the adherent cells were detached by mechanical scraping, the mammalian cells were lysed by mechanical force (Bullet Blender, NextAdvance USA, power 8 for 1 min) and the bacteria were filtered using a 2 μm filter.

### 4.2 Quantification of bacterial growth by qPCR

The effect of dyes on bacterial viability was measured by growing bacteria in L929 cells in 24-well tissue culture plates (Corning, USA) in the presence or absence of dyes or mock-labelling conditions. Identical triplicate wells were used. After 7 days growth at 35 °C + 5% CO_2_, bacterial DNA was isolated and the genome copy number was determined using qPCR against the STA47 gene (OTT_1319) as described previously (Giengkam et al., 2015). Results are shown as the absolute bacterial genome copy number per well of a 24 well plate (1.9 cm^2^) and the inoculum added per well is given in the figure legend. Note that the inoculum is not the same as the number of bacteria taken up by cells, which is typically 1–100 times lower. Unbound bacteria were washed away 3 h after inoculation and prior to incubation for 7 days.

### 4.3 Immunofluorescence labelling and confocal microscopy

For live imaging or fixed sample preparation of bacteria inside L929 host cells, L929 cells were grown directly on chambered coverslip slides (Ibidi, USA). These were infected with labelled or unlabelled bacteria and either imaged live or fixed and processed for labelling with fluorescently-conjugated antibodies. Samples were fixed with 4% paraformaldehyde for 10 min at room temperature, and then washed three times with PBS. Samples were permeabilised using 0.5% triton X on ice for ten minutes, and then washed three times with PBS. Immunofluorescent labelling was performed using a rat monoclonal antibody against the TSA56 gene diluted to 1/200, followed by an alexa 488-conjugated secondary anti-rat antibody diluted to 1/1000 (ThermoFisher Scientific, UK). Incubations were performed for 30–60 min at room temperature, and washed three times with PBS in between. Samples were mounted using hardset mounting media (Vectashield, Vector Laboratories, USA). Imaging was performed using a Zeiss LSM 7000 equipped with a 63 × 1.4 NA objective lens (Carl Zeiss, USA).

### 4.4 CFSE and CellTrace FarRed labelling

Purified bacteria were labelled with CFSE and CT FarRed as follows (catalog numbers C34554 and C34564 from LifeTechnologies, USA). 5 mM aliquots of dye dissolved in DMSO were stored in 1 μl volumes at − 20 °C. For labelling, one aliquot was thawed to room temperature. This was diluted to a 5 μM working dilution in PBS containing 2% BSA. Purified bacteria were pelleted at 20,238 ×* g* at room temperature, then resuspended in the 5 μM dye solution. Samples were incubated for 15 min at 37 °C or 30 min at room temperature, in the dark. Bacteria were then pelleted and resuspended in RPMI + 5% FBS and incubated at room temperature for 30 min to quench remaining dye. Bacteria were repelleted and resuspended in fresh media, and were then ready for use.

### 4.5 CFSE quantification

CFSE-labelled bacteria were added to growing L929 cells or cell-free media, in chambered coverglass slides, then imaged by confocal microscopy at various times up to 7 days. At each time point, images were recorded using identical laser strength and gain parameters. Ten bacteria were randomly selected and the maximum pixel intensity within the bacteria was recorded. This experiment was repeated three independent times and the same trend observed each time.

### 4.6 SYTO9 labelling

The dye SYTO9 (catalog number S34854, Invitrogen, USA) was dissolved to 5 μM in DMSO and stored in 5 μl aliquots at − 20 °C. Purified bacteria or bacteria in L929 cells were labelled with SYTO9 by adding the dye directly to samples at a dilution of 1/1000, which was equivalent to 5 nM.

### 4.7 Vybrant dye labelling

The Vybrant dyes DiI, DiD and DiO (catalog number V22889, LifeTechnologies, USA) were used to label purified bacteria. The dyes were stored in their commercial buffers at 4 °C. Purified bacteria were resuspended in 100 μl of the commercial Opti-MEM buffer (catalog number 31985062, ThermoFisher Scientific, UK), and the dye was added at a dilution of 1/100 (final concentration of 100 μM). Samples were incubated at 35 °C for 30 min, then washed by pelleting two times and then resuspended in DMEM + 5% FBS.

### 4.8 HADA labelling

HADA was used to label purified bacteria or bacteria growing in L929 cells. HCC-amino-D-alanine (HADA, synthesised by Michael Vannieuwenhze, University of Indiana, USA) was stored in 5 μl aliquots at 10 mM in DMSO at − 20 °C. For labelling, HADA was dissolved in growth media at a dilution of 1/100 (final concentration of 100 μM). Bacteria were washed and resuspended in HADA + growth media (to label purified bacteria) or the media was removed and replace with HADA + growth media (bacteria in L929 cells). Samples were incubated at 35 °C for 3 h, then either imaged live or washed three times in PBS and fixed before imaging.

### 4.9 Statistical analysis

Statistical analysis was performed using GraphPad Prism software (GraphPad). One-way ANOVA analysis was used.

The following are the supplementary data related to this article.

Movie 1 CFSE-labelled bacteria entering L929 cells. Scale bar = 10 μM. Time elapsed is shown in the top left hand corner. Movie 1

Movie 2 SYTO9-labelled bacteria inside L929 cells. Scale bar = 10 μM. Time elapsed is shown in the top left hand corner. Movie 2

Movie 3 HADA-labelled bacteria entering L929 cells. Scale bar = 10 μM. Time elapsed is shown in the top left hand corner. Movie 3

Supplementary Fig. 1 Graph showing the growth of *Orientia* in L929 cells over 7 days. Prior to infection, bacteria were treated with no label, mock label or CT FarRed label. Bacterial copy number per well was determined by qPCR and biological triplicate values are plotted. Supplementary Fig. 1

## Glossary

HADA: HCC-amino-d-alanine
CFSE: carboxyfluorescein succinimidyl ester
CFDA-SE: carboxyfluorescein diacetate succinimidyl ester

## Figures and Tables

**Table 1:** Summary of fluorescent probes used in this study.

Fluorescent probe	Excitation max. (nm)/Emission max. (nm)	Labelling target	Fixable?	Effect on growth	Processes particularly amenable to study
CFSE, CT FarRed	492/517; 630/661	Intracellular amines	Poorly	None	Bacterial attachment and entry into host cells; cell division by dye dilution
DiO; DiI; DiD	484/501; 549/565; 644/665	Lipid bilayers	No	Significant	Bacterial attachment and entry into host cells; generation of outer membrane vesicles; bacterial shape and growth
Syto9	485/498	DNA and RNA	No	Minimal	Bacterial motility
HADA	405/460	Peptidoglycan	Yes	None	Bacterial attachment and entry into host cells; cell wall; bacterial shape and growth

## References

[bb0005] Amano K. (1987). Deficiency of peptidoglycan and lipopolysaccharide components in *Rickettsia tsutsugamushi*. Infect. Immun..

[bb0010] Belin B.J., Goins L.M., Mullins R.D. (2014). Comparative analysis of tools for live cell imaging of actin network architecture. BioArchitecture.

[bb0015] Betz W.J., Bewick G.S. (1992). Optical analysis of synaptic vesicle recycling at the frog neuromuscular junction. Science.

[bb0020] Boleti H., Ojcius D.M., Dautry-Varsat A. (2000). Fluorescent labelling of intracellular bacteria in living host cells. J. Microbiol. Methods.

[bb0025] Bronner-Fraser M. (1985). Alterations in neural crest migration by a monoclonal antibody that affects cell adhesion. J. Cell Biol..

[bb0030] Brown D.A., London E. (1998). Functions of lipid rafts in biological membranes. Annu. Rev. Cell Dev. Biol..

[bb0035] Giengkam S. (2015). Improved quantification, propagation, purification and storage of the obligate intracellular human pathogen *Orientia tsutsugamushi*. PLoS Negl. Trop. Dis..

[bb0040] Helaine S. (2010). Dynamics of intracellular bacterial replication at the single cell level. Proc. Natl. Acad. Sci. U. S. A..

[bb0045] Helaine S. (2014). Internalization of Salmonella by macrophages induces formation of nonreplicating persisters. Science.

[bb0050] Kuru E., Tekkam S., Hall E., Brun Y.V., Van Nieuwenhze M.S. (2015). Synthesis of fluorescent d-amino acids and their use for probing peptidoglycan synthesis and bacterial growth in situ. Nat. Protoc..

[bb0055] Landgraf D., Okumus B., Chien P., Baker T.A., Paulsson J. (2012). Segregation of molecules at cell division reveals native protein localization. Nat. Methods.

[bb0060] Maurer J.A., Dougherty D.A. (2001). A high-throughput screen for MscL channel activity and mutational phenotyping. Biochim. Biophys. Acta.

[bb0065] Moree M.F., Hanson B. (1992). Growth characteristics and proteins of plaque-purified strains of *Rickettsia tsutsugamushi*. Infect. Immun..

[bb0070] Nose A., Takeichi M. (1986). A novel cadherin cell adhesion molecule: its expression patterns associated with implantation and organogenesis of mouse embryos. J. Cell Biol..

[bb0075] Obradovic M., Pasternak J.A., Hon Ng S., Wilson H.L. (2016). Use of flow cytometry and PCR analysis to detect 5′-carboxyfluoroscein-stained obligate intracellular bacteria *Lawsonia intracellularis* invasion of McCoy cells. J. Microbiol. Methods.

[bb0080] Paris D.H., Shelite T.R., Day N.P., Walker D.H. (2013). Unresolved problems related to scrub typhus: a seriously neglected life-threatening disease. Am.J.Trop. Med. Hyg..

[bb0085] Parish C.R. (1999). Fluorescent dyes for lymphocyte migration and proliferation studies. Immunol. Cell Biol..

[bb0090] Parish C.R., Warren H.S. (2002). Use of the intracellular fluorescent dye CFSE to monitor lymphocyte migration and proliferation. Curr Protoc Immunol.

[bb0095] Peng T. (2010). Determining the distribution of probes between different subcellular locations through automated unmixing of subcellular patterns. Proc. Natl. Acad. Sci. U. S. A..

[bb0100] Pogliano J. (1999). A vital stain for studying membrane dynamics in bacteria: a novel mechanism controlling septation during *Bacillus subtilis* sporulation. Mol. Microbiol..

[bb0105] Pol A. (2001). A caveolin dominant negative mutant associates with lipid bodies and induces intracellular cholesterol imbalance. J. Cell Biol..

[bb0110] Rajapakse S., Rodrigo C., Fernando D. (2012). Scrub typhus: pathophysiology, clinical manifestations and prognosis. Asian Pac J Trop Med.

[bb0115] Seong S.Y., Choi M.S., Kim I.S. (2001). *Orientia tsutsugamushi* infection: overview and immune responses. Microbes Infect..

[bb0120] Shaner N.C. (2008). Improving the photostability of bright monomeric orange and red fluorescent proteins. Nat. Methods.

[bb0125] Shieh P., Siegrist M.S., Cullen A.J., Bertozzi C.R. (2014). Imaging bacterial peptidoglycan with near-infrared fluorogenic azide probes. Proc. Natl. Acad. Sci. U. S. A..

[bb0130] The Molecular Probes Handbook, *Eleventh Edition*. n.d.

[bb0135] Tuominen-Gustafsson H., Penttinen M., Hytonen J., Viljanen M.K. (2006). Use of CFSE staining of borreliae in studies on the interaction between borreliae and human neutrophils. BMC Microbiol..

[bb0140] Turner R.D., Hurd A.F., Cadby A., Hobbs J.K., Foster S.J. (2013). Cell wall elongation mode in Gram-negative bacteria is determined by peptidoglycan architecture. Nat. Commun..

[bb0145] Turner R.D., Vollmer W., Foster S.J. (2014). Different walls for rods and balls: the diversity of peptidoglycan. Mol. Microbiol..

[bb0150] Vollmer W., Blanot D., de Pedro M.A. (2008). Peptidoglycan structure and architecture. FEMS Microbiol. Rev..

[bb0155] Watt G., Parola P. (2003). Scrub typhus and tropical rickettsioses. Curr. Opin. Infect. Dis..

