# Supplementary figures and images for: Live imaging of the genetically intractable obligate intracellular bacteria Orientia tsutsugamushi using a panel of fluorescent dyes
Source: J Microbiol Methods. 2016 Nov;130:169–76. doi: 10.1016/j.mimet.2016.08.022 (PMC5073074; doi:10.1016/j.mimet.2016.08.022)

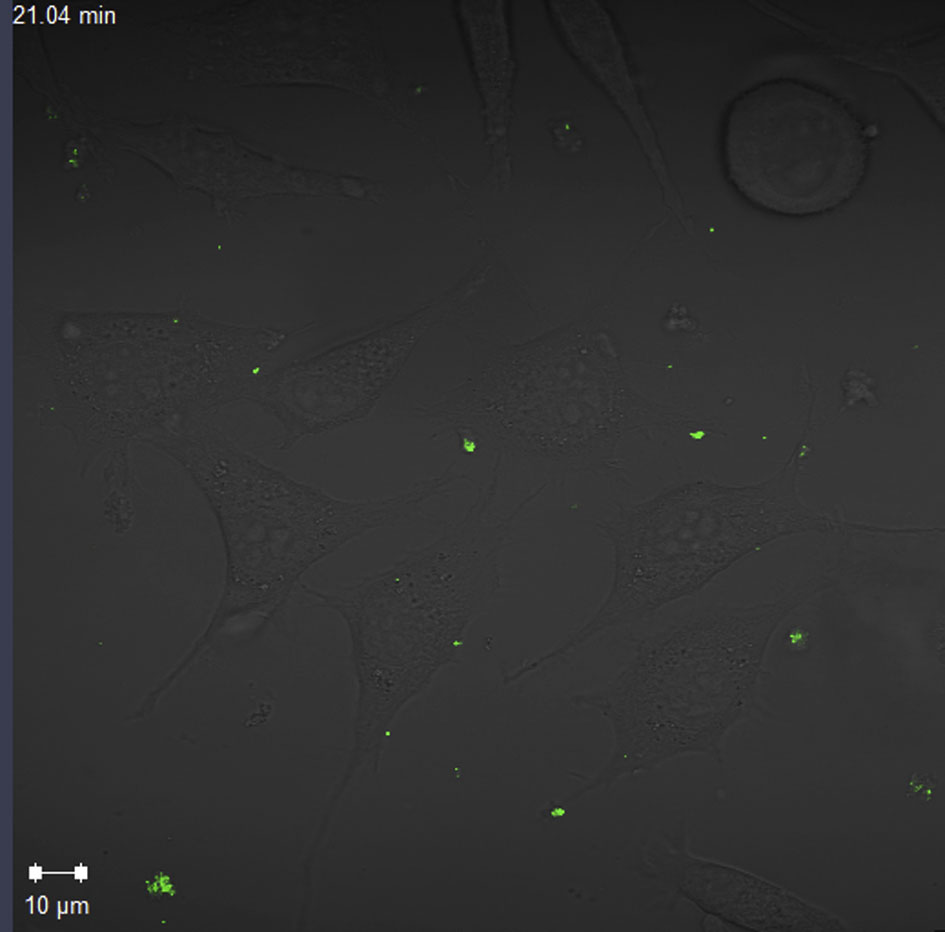

Supplement: Movie 1 — CFSE-labelled bacteria entering L929 cells. Scale bar = 10 μM. Time elapsed is shown in the top left hand corner. [file mmc1.jpg]

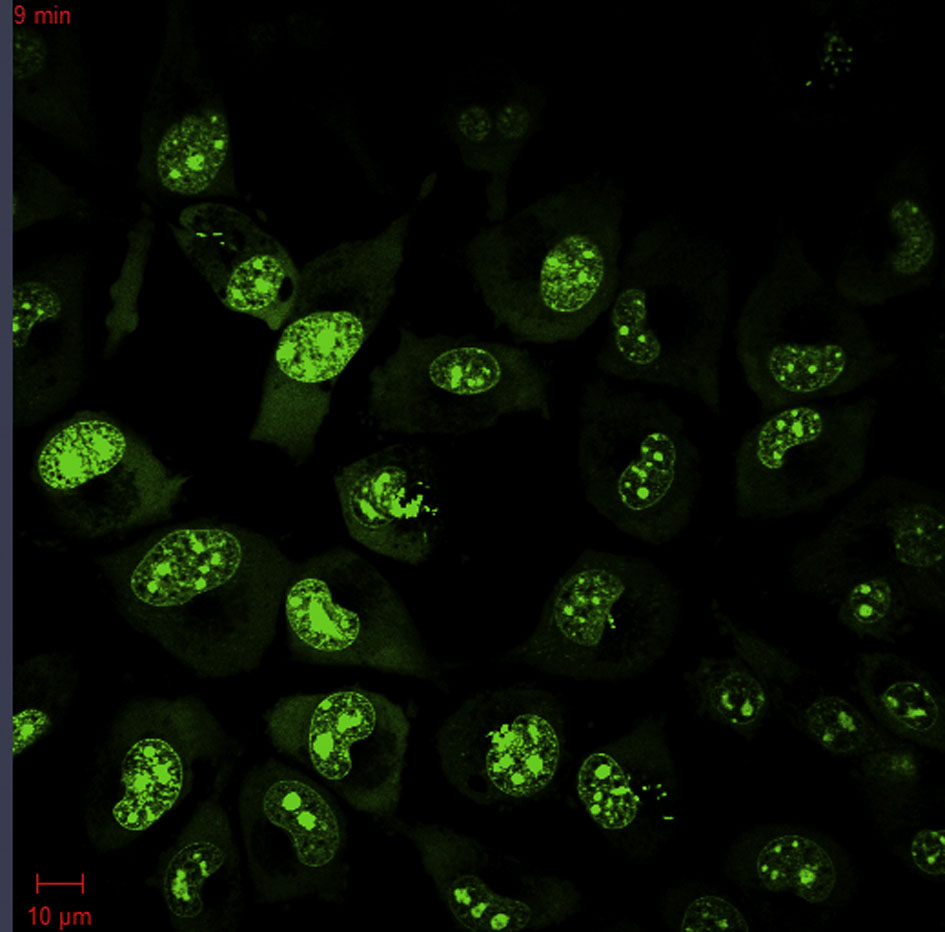

Supplement: Movie 2 — SYTO9-labelled bacteria inside L929 cells. Scale bar = 10 μM. Time elapsed is shown in the top left hand corner. [file mmc2.jpg]

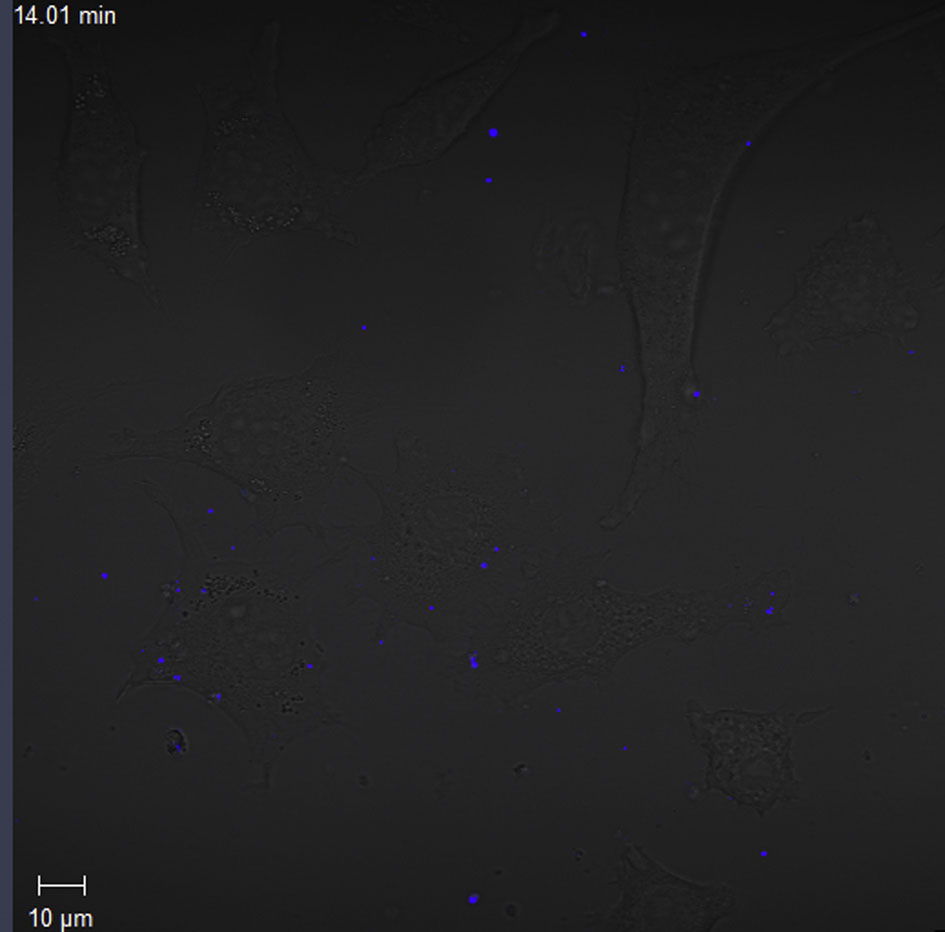

Supplement: Movie 3 — HADA-labelled bacteria entering L929 cells. Scale bar = 10 μM. Time elapsed is shown in the top left hand corner. [file mmc3.jpg]
