# Supplementary material for: Live imaging of the genetically intractable obligate intracellular bacteria Orientia tsutsugamushi using a panel of fluorescent dyes
Source: J Microbiol Methods. 2016 Nov;130:169–76. doi: 10.1016/j.mimet.2016.08.022 (PMC5073074; doi:10.1016/j.mimet.2016.08.022)
Supplement: Supplementary Fig. 1 — Graph showing the growth of Orientia in L929 cells over 7 days. Prior to infection, bacteria were treated with no label, mock label or CT FarRed label. Bacterial copy number per well was determined by qPCR and biological triplicate values are plotted. [file mmc4.pdf]

Supplementary Figure 1. Growth curve of Orientia tsutsugamushi in L929 cells

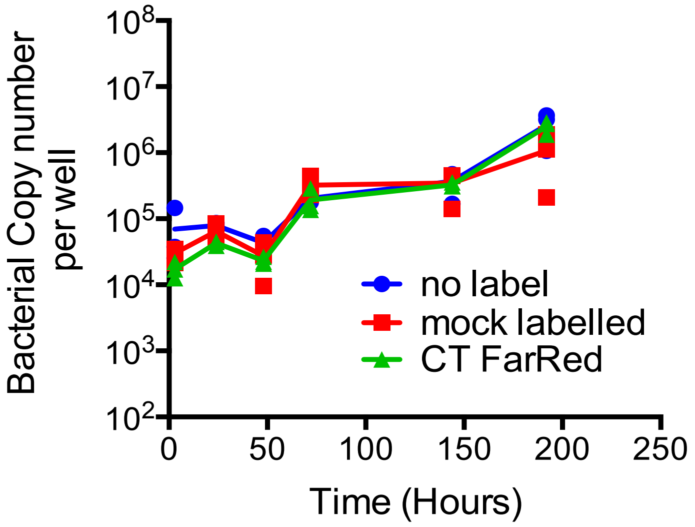

Supplementary Figure 1. Graph showing the growth of Orientia in L929 cells over 7 days. Prior to infection, bacteria were treated with no label, mock label or CT FarRed label. Bacterial copy number per well was determied by qPCR and biological triplicate values are plotted.
